# Supplementary material for: Rational Design of Mono- and Bi-Nuclear Cyclometalated Ir(III) Complexes Containing Di-Pyridylamine Motifs: Synthesis, Structure, and Luminescent Properties
Source: Molecules. 2022 Sep 15;27(18):6003. doi: 10.3390/molecules27186003 (PMC9503406; doi:10.3390/molecules27186003)
Supplement: Supplementary file 1 [file molecules-27-06003-s001.zip › molecules-1835599-supplementary.pdf]

Supporting Information

Rational Design of Mono- and Bi-nuclear Cyclometalated Ir(III) Complexes Containing Di-pyridylamine Motifs: Synthesis, Structure and Luminescent Properties

Hugo Sesolis [1], Geoffrey Gontard [1], Marie Noelle Rager [2], Elisa Bandini [3], Alejandra Saavedra Moncada [3], Andrea Barbieri [3,]* and Hani Amouri [1,]*

[1] Sorbonne Universitét Campus Pierre et Marie Curie, Institut Parisien de Chimie Moléculaire (IPCM) UMR CNRS 8232, 4 place Jussieu, 75252 Paris cedex 05, Franc
[2] Chimie ParisTech, PSL University, NMR Facility, Ft 75005 Paris, France
[3] Istituto per la Sintesi Organica e la Fotoreattività (ISOF) Consiglio Nazionale delle Ricerche (CNR) Via Gobetti 101, 40129 Bologna, Italy

* Correspondence: AB: andrea.barbieri@isof.cnr.it HA : hani.amouri@sorbonne-universite.fr

TABLE OF CONTENTS

Figures S1-S8 (^{1}H, ^{13}C) -NMR spectra of compounds (**1-4**).

Figure S9 HRMS (ESI) spectra of complexes **3** and **4**.

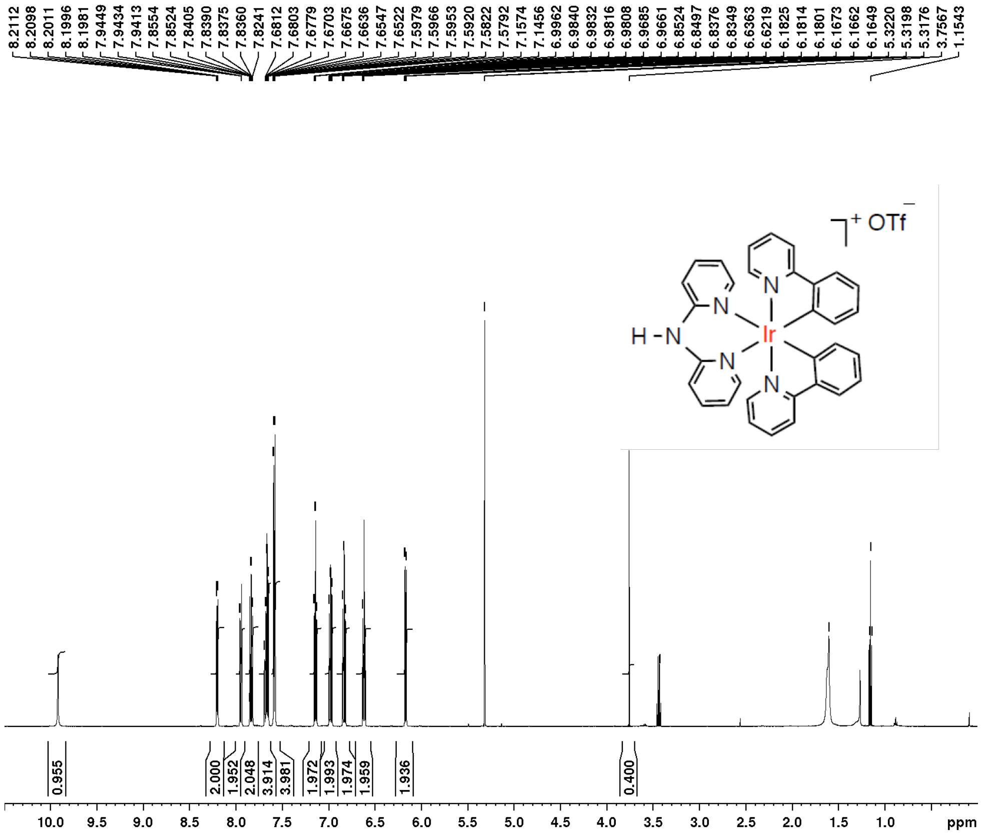

Figure S1. ^{1}H NMR (500 MHz, CD_2Cl_2) of compound **1**

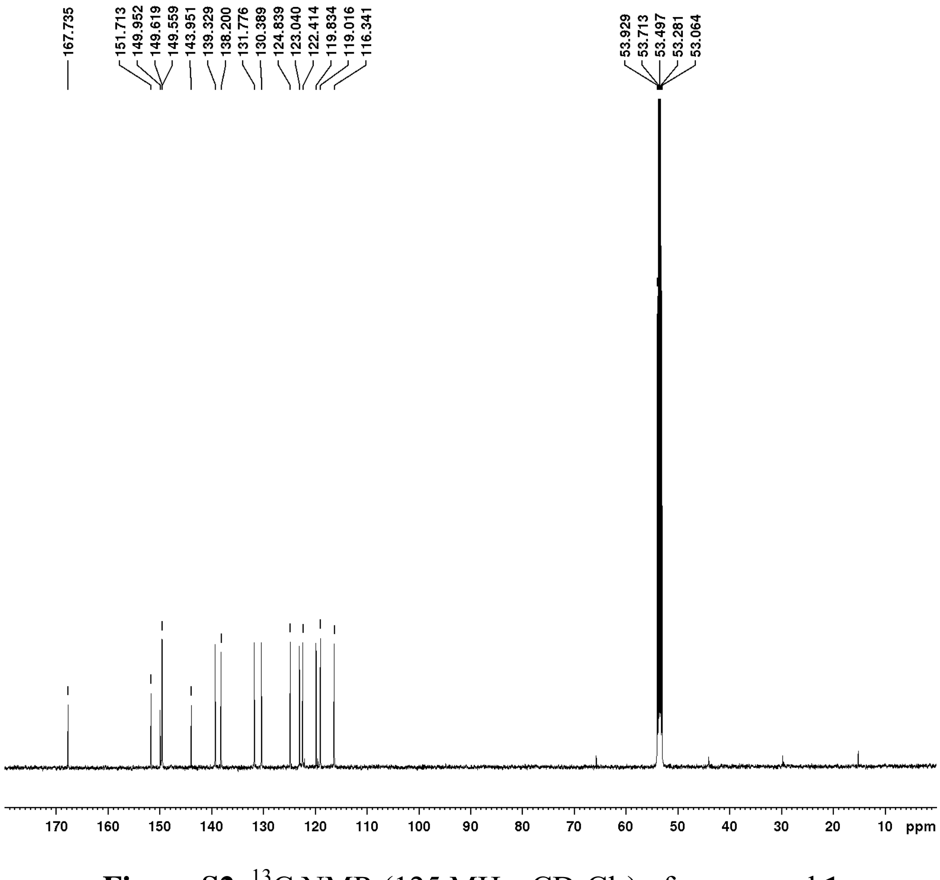

Figure S2. ^{13}C NMR (125 MHz, CD_2Cl_2) of compound **1**

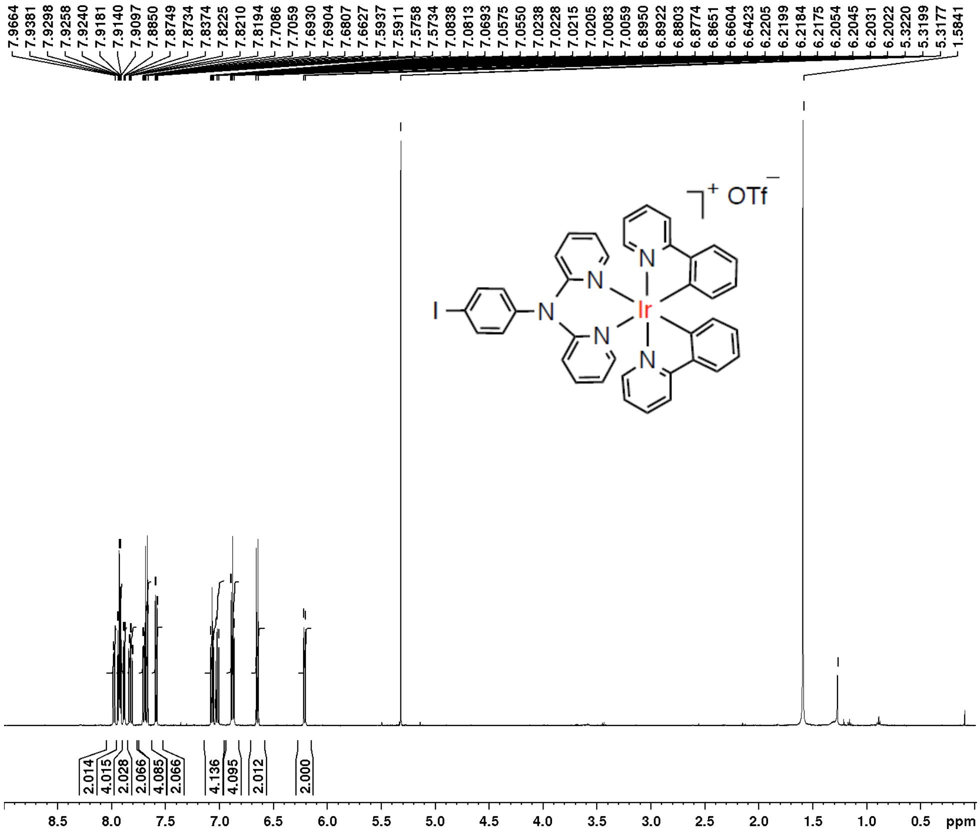

Figure S3. ^{1}H NMR (500 MHz, CD_2Cl_2) of compound **2**

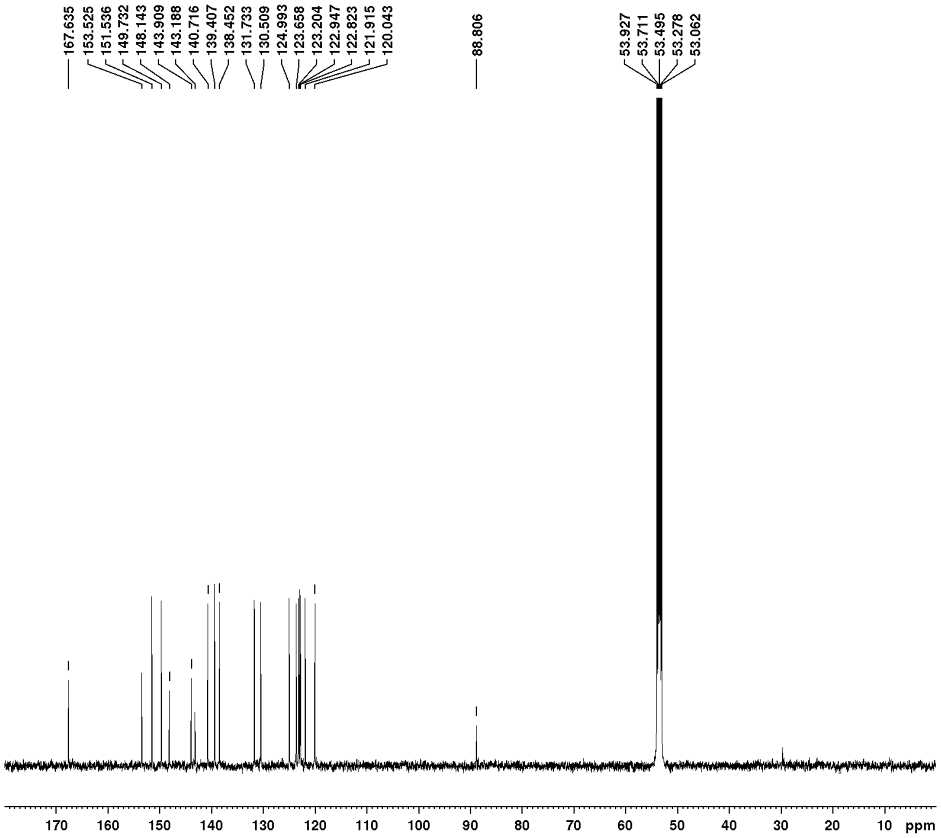

Figure S4. ^{13}C NMR (125 MHz, CD_2Cl_2) of compound **2**

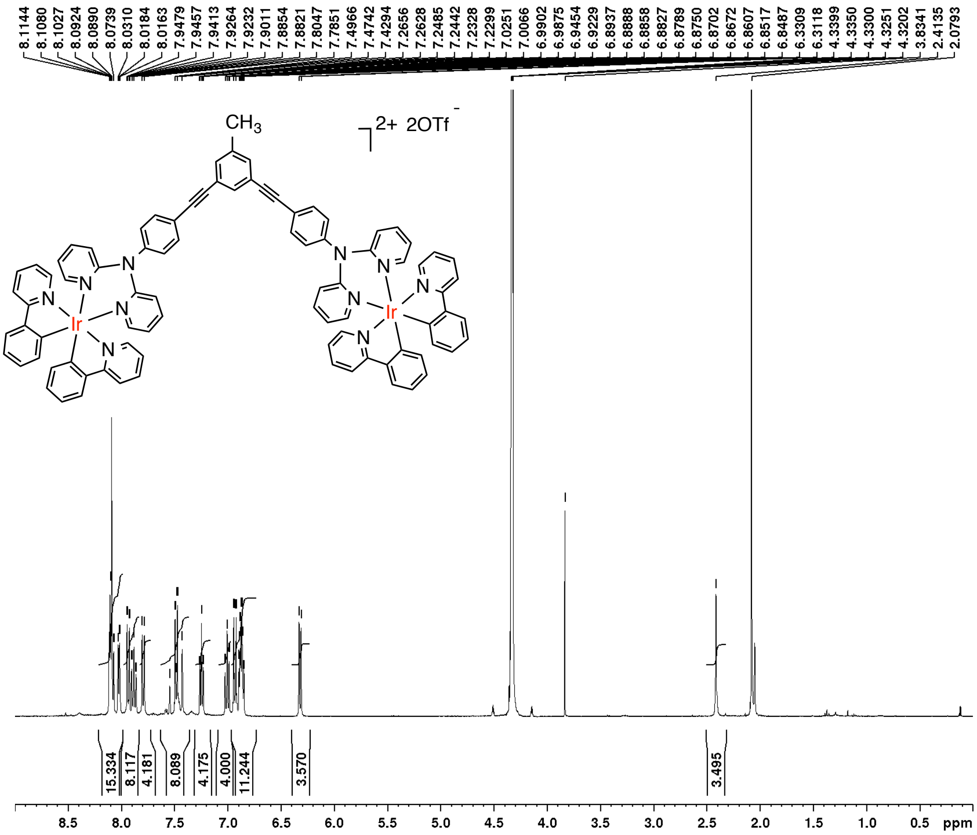

Figure S5. ^{1}H NMR (400 MHz, CD_3NO_2) of compound **3**

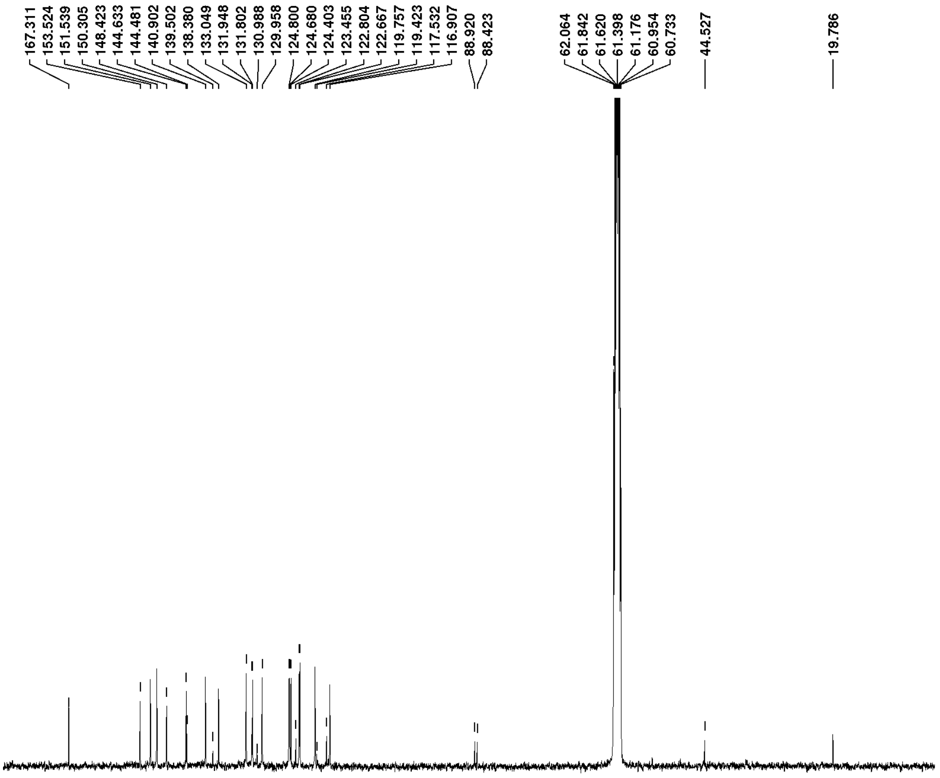

Figure S6. ^{13}C NMR (100 MHz, CD_3NO_2) of compound **3**

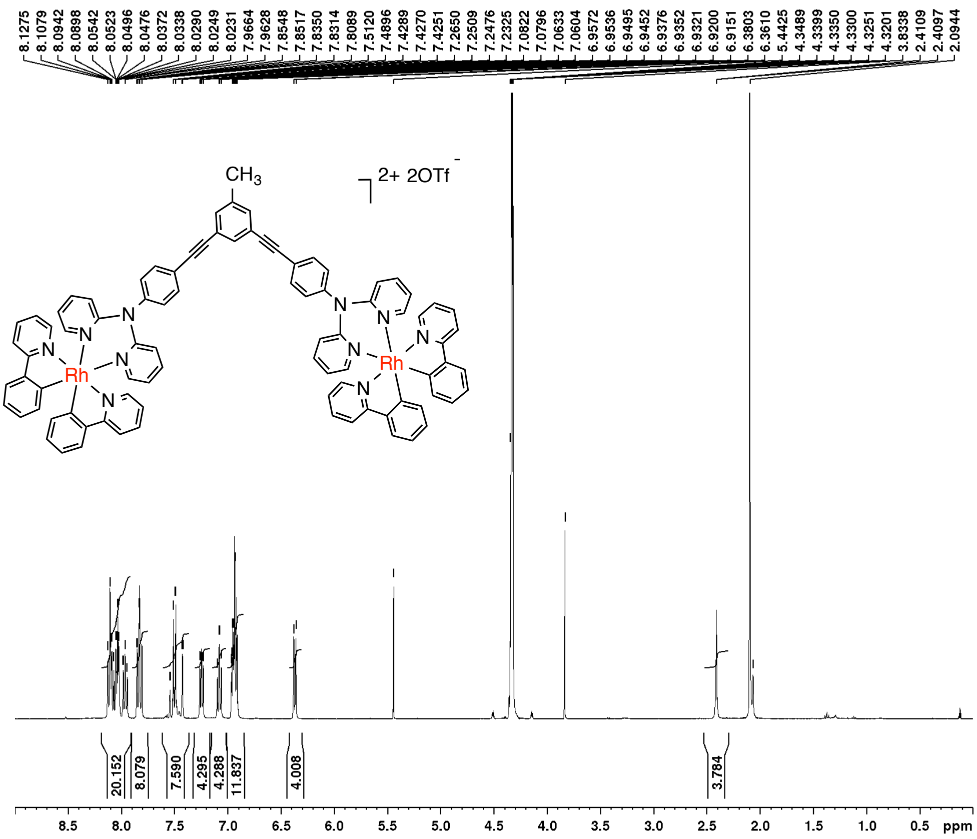

Figure S7. ^{1}H NMR (400 MHz, CD_3NO_2) of compound **4**

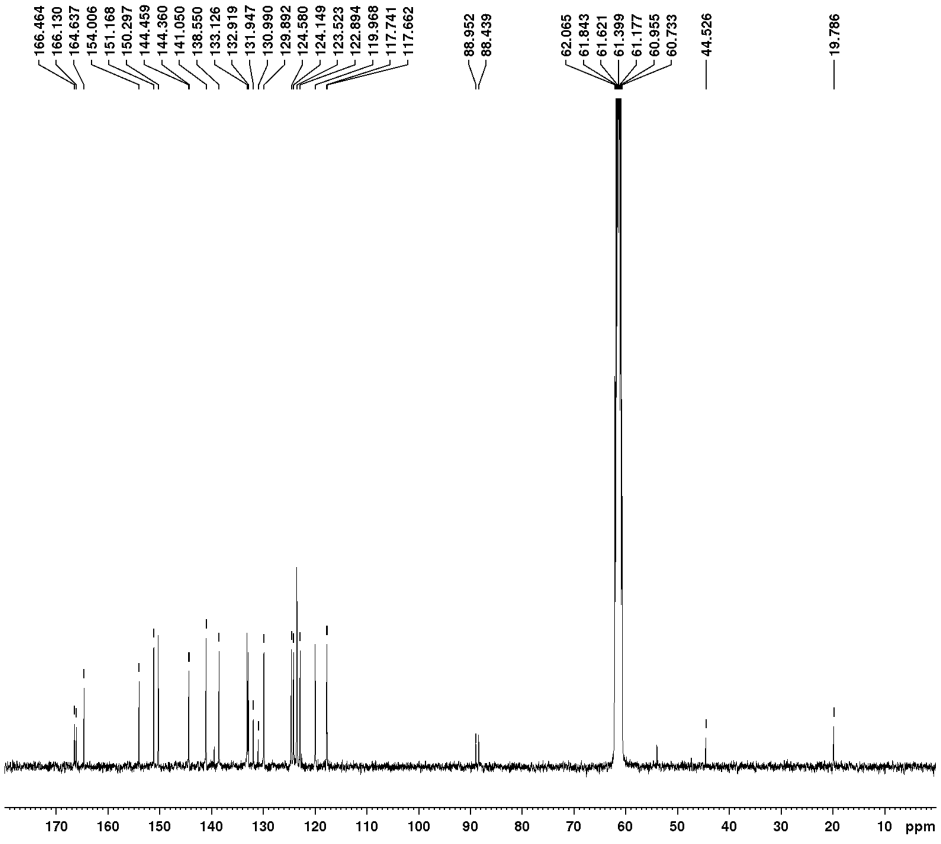

Figure S8. ^{13}C NMR (100 MHz, CD_3NO_2) of compound **4**

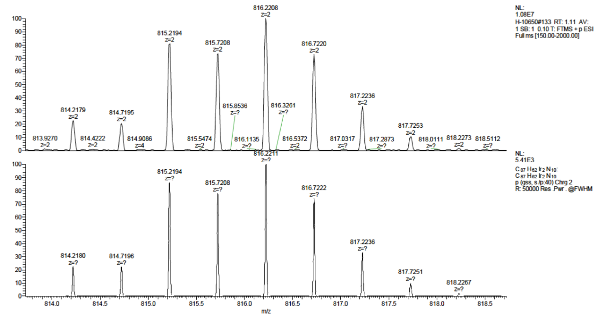

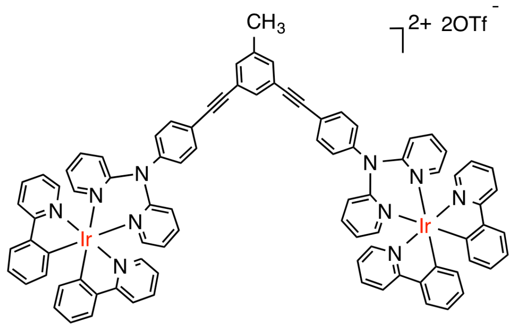

Experimental/theoretical isotopic pattern MS spectrum

Error = 0.6 ppm; Relative Intensity (%) 100

HRMS (ESI) m/z: [M]2+ Calcd for C87H62Ir2N10 816.2203 . Found 816.2208; (Error: 0.6 ppm).

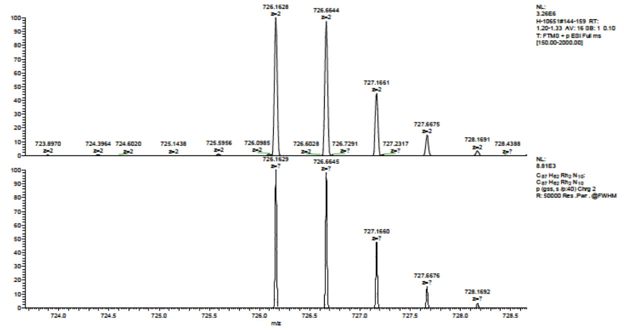

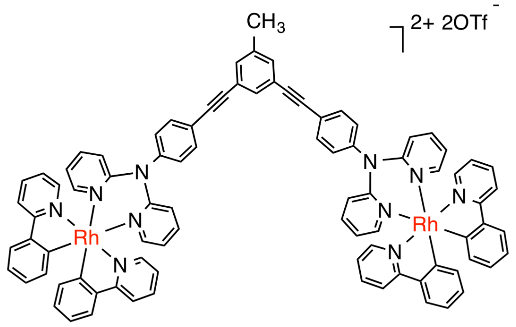

Experimental/theoretical isotopic pattern MS spectrum

Error = -0.1 ppm; Relative Intensity (%) 100

HRMS (ESI) m/z: [M]2+ Calcd for C87H62Rh2N10 726.1629 . Found 726.1628; (Error: -0.1 ppm).

Figure S9. HRMS (ESI) spectra of complexes **3** and **4**.
